# Association between non-high-density lipoprotein cholesterol to high-density lipoprotein cholesterol ratio and age-related macular degeneration: insights from two observational studies

**DOI:** 10.3389/fmed.2025.1724938

**Published:** 2025-12-10

**Authors:** Xin Chen, Haiyu Li, Ziman Jiao, Yuxin Liu, Yuting Wu, Xinyue Qiu, Yuelan Zou, Guanghui Liu

**Affiliations:** 1 The Affiliated People’s Hospital of Fujian University of Traditional Chinese Medicine (Fujian Provincial People’s Hospital), Fuzhou, China; 2 Eye Institute of Integrated Chinese and Western Medicine, Fujian University of Traditional Chinese Medicine, Fuzhou, China; 3 Shanghai Eye Disease Prevention and Treatment Center, Shanghai, China

**Keywords:** NHHR, age-related macular degeneration, National Health and Nutrition Examination Surveys, lipid metabolism, cross-sectional analysis, retrospective study

## Abstract

**Objectives:**

The non-high-density lipoprotein cholesterol to high-density lipoprotein cholesterol ratio (NHHR) is an innovative measure for assessing cardiovascular disease risk, primarily associated with lipid profiles. Lipid metabolism disorders have been reported to be associated with age-related macular degeneration (AMD), yet the relationship between NHHR and AMD has not been previously explored. This study primarily aims to investigate the potential association between NHHR and the prevalence of AMD.

**Methods:**

A comprehensive cross-sectional stratified survey using the National Health and Nutrition Examination Survey (NHANES) dataset of the US was conducted, including 4,017 participants aged 40 years and older, from 2005 to 2008. The NHHR was calculated as [Total Cholesterol (TC) − High-Density Lipoprotein Cholesterol (HDL-C)]/HDL-C. Data on AMD were derived from retinal photography. Logistic regression, stratified analysis, RCS curve, ROC/AUC, and subgroup interaction analysis were used to explore the relationship between NHHR and AMD. Meanwhile, patients who visited the People’s Hospital Affiliated to Fujian University of Traditional Chinese Medicine (PHFT, China) between November 2021 and September 2025 were recruited retrospectively. All participants who met the study inclusion criteria were screened from the hospital-wide integrated informatics platform for clinical research. Finally, the clinical data of 96 eligible participants of PHFT were included in this study for conducting external validation analysis.

**Results:**

The cross-sectional study from NHANES included 4,017 participants, of whom 3,678 (91.56%) had no AMD and 339 (8.44%) exhibited AMD. The external validation study from PHFT consisted of 48 patients with AMD and 48 non-AMD participants. Both studies indicated that compared with the non-AMD group, the AMD group had a significantly lower NHHR (*p* < 0.01). In the fully adjusted Model, when NHHR was stratified into tertiles, the results showed that for each one-unit increase in NHHR, the risk of AMD in individuals in the highest tertile was reduced by 33.1 and 76.8%, respectively. The results of the RCS curve and threshold effect analyses from the two studies confirmed a negative correlation trend between the two variables (*p* < 0.05). The subgroup and interaction analysis, based on data from the NHANES, shows consistent associations between NHHR and AMD across various subgroups.

**Conclusion:**

Our preliminary research indicates that NHHR might be a reliable independent indicator of the risk of developing AMD. In the future, large-scale sample studies and more prospective research are still needed to confirm our findings.

## 1 Introduction

Age-related macular degeneration (AMD) is a leading irreversible cause of severe vision loss. This disease primarily affects the macular region in the retina, causing progressive central vision loss that significantly impairs patients’ quality of life. A substantial proportion of elderly individuals (typically aged >60 years) experience significant visual impairment due to AMD (1, 2). Studies indicate that AMD prevalence increases from 0.3 per 1,000 in adults aged 55–59 years to 36.7 per 1,000 in those ≥90 years. Epidemiological data predict that AMD will affect approximately 288 million people worldwide by 2040 (3). AMD poses a serious global threat to visual health in middle-aged and elderly populations, with prevalence rates rising steadily due to population aging. Therefore, in-depth research on AMD’s pathological mechanisms and therapeutic interventions is crucial.

Dry AMD (dAMD), the most prevalent form, is characterized by progressive atrophy of the retinal pigment epithelium (RPE), photoreceptors, and choroidal capillaries in the macula (4). Drusen deposition is a hallmark feature of AMD. Extensive drusen deposits between the RPE cells and Bruch’s membrane form a physical barrier. Some studies hypothesize that this barrier may induce chronic hypoxia and nutrient deficiency, leading to the gradual deterioration of RPE cell function (5), which in turn could trigger geographic atrophy (GA) and ultimately might result in central vision loss. Often arising from dAMD, wet AMD (wAMD) exhibits rapid progression despite its lower prevalence. It is characterized by choroidal neovascularization (CNV) beneath the RPE layer. These fragile vessels frequently leak and hemorrhage, resulting in acute vision loss. Clinically, AMD is categorized into early and late stages based on disease progression and severity. Early AMD primarily presents with scattered drusen and mild RPE abnormalities, while late AMD involves GA and CNV formation (2). AMD pathogenesis is multifactorial, involving genetic predisposition, environmental factors, oxidative stress, and chronic inflammation. Current AMD treatments include laser therapy, anti-VEGF agents, complement inhibitors, RPE transplantation, stem cell therapy, and gene therapy. Laser therapy and anti-VEGF agents are the primary clinical interventions. However, laser therapy fails to inhibit neovascularization and may cause collateral retinal damage. Anti-VEGF therapy is mainly used for the treatment of wAMD, though it requires long-term and repeated administration of medications and is associated with high costs. For dAMD, no effective treatment currently exists (6). To improve AMD prevention and monitoring, developing reliable diagnostic tools is essential.

Current AMD diagnostic methods exhibit limited detection accuracy. Commonly used examinations include fundus photography, optical coherence tomography (OCT), and fundus fluorescein angiography (FFA). Ophthalmologists rely on these images to confirm the diagnosis. However, the examination results may have errors due to the technical differences among operators and the limitations of the machines. Furthermore, the diagnostic workflow is both costly and time-consuming (7), and these examination methods may lack sensitivity in detecting AMD, leading to some patients not being diagnosed, making the discovery of new AMD-related bioindicators crucial for the prevention and treatment.

Substantial evidence links AMD pathogenesis with dysregulated lipid metabolism (8–10). Lipids are not only important cellular components but also essential energy sources, maintaining the normal physiological functions of the retina. Dysregulation of lipid metabolism triggers lipid deposition in the retinal pigment epithelium and Bruch’s membrane, leading to drusen formation and ultimately resulting in photoreceptor atrophy. As a barrier between the RPE and choroid, the long-term presence of these deposits in Bruch’s membrane hinders nutrient exchange, promoting atrophy of the RPE and choroid. Simultaneously, it triggers inflammatory cascades and oxidative stress, accelerating the progressive loss of photoreceptors, disrupting the tight junctions of the RPE, impairing its barrier function, and inducing fluid leakage, thereby accelerating the progression of AMD (11, 12). Recently, serum biomarkers have emerged as promising diagnostic tools (13). The diagnosis and classification of AMD primarily rely on retinal imaging techniques currently. Although these methods are indispensable, they mainly detect structural changes that typically manifest at intermediate or late stages of the disease. There is a critical need for an objective and accessible tool capable of identifying high-risk individuals before significant retinal damage occurs, thereby enabling early intervention. Serum biomarkers, which often reflect systemic biochemical alterations, have emerged as a promising solution. Among the various pathophysiological pathways implicated in AMD, dysregulation of lipid metabolism has attracted considerable attention. Serum lipid profiling offers a convenient, cost-effective approach for AMD risk assessment. The non-high-density lipoprotein cholesterol (non-HDL-C) to high-density lipoprotein cholesterol ratio (HDL-C) is a new type of composite lipid ratio, which was introduced by Chinese scholars in 2022 following a longitudinal study involving 15,000 individuals (14, 15). Calculated as NHHR = non-HDL-C/HDL-C, it includes factors potentially associated with dyslipidemia, namely non-HDL-C and HDL-C (16). It can more comprehensively reflect a patient’s lipid health status compared to single lipid indicators and has broad clinical predictive value. To date, the relationship between NHHR and AMD has not been studied. In this study, first, the potential association between NHHR and AMD was analyzed based on the data from the National Health and Nutrition Examination Survey (NHANES) database. Subsequently, the data of subjects from the People’s Hospital Affiliated to Fujian University of Traditional Chinese Medicine (PHFT, China) were evaluated to verify the reliability and practicality of the above-mentioned results. The aim is to clarify the association between the two and provide a reference for clinical prevention and control.

## 2 Materials and methods

### 2.1 Study participants in NHANES

This cross-sectional study extracted data from the NHANES database from 2005 to 2008 for analysis. NHANES is a nationally representative survey assessing the health and nutritional status of the non-institutionalized US population. It employs a stratified, multistage probability sampling design to ensure national representativeness. The information on sociodemographic, lifestyle, dietary intake, behaviors, and medical conditions collected through questionnaires or physical examinations can help researchers evaluate the relationships between various factors and diseases (17). The project passed the ethical approval of a human subject’s board and obtained written informed consent from all participants. All procedures follow the principles of the Declaration of Helsinki (18).

**Figure 1:**
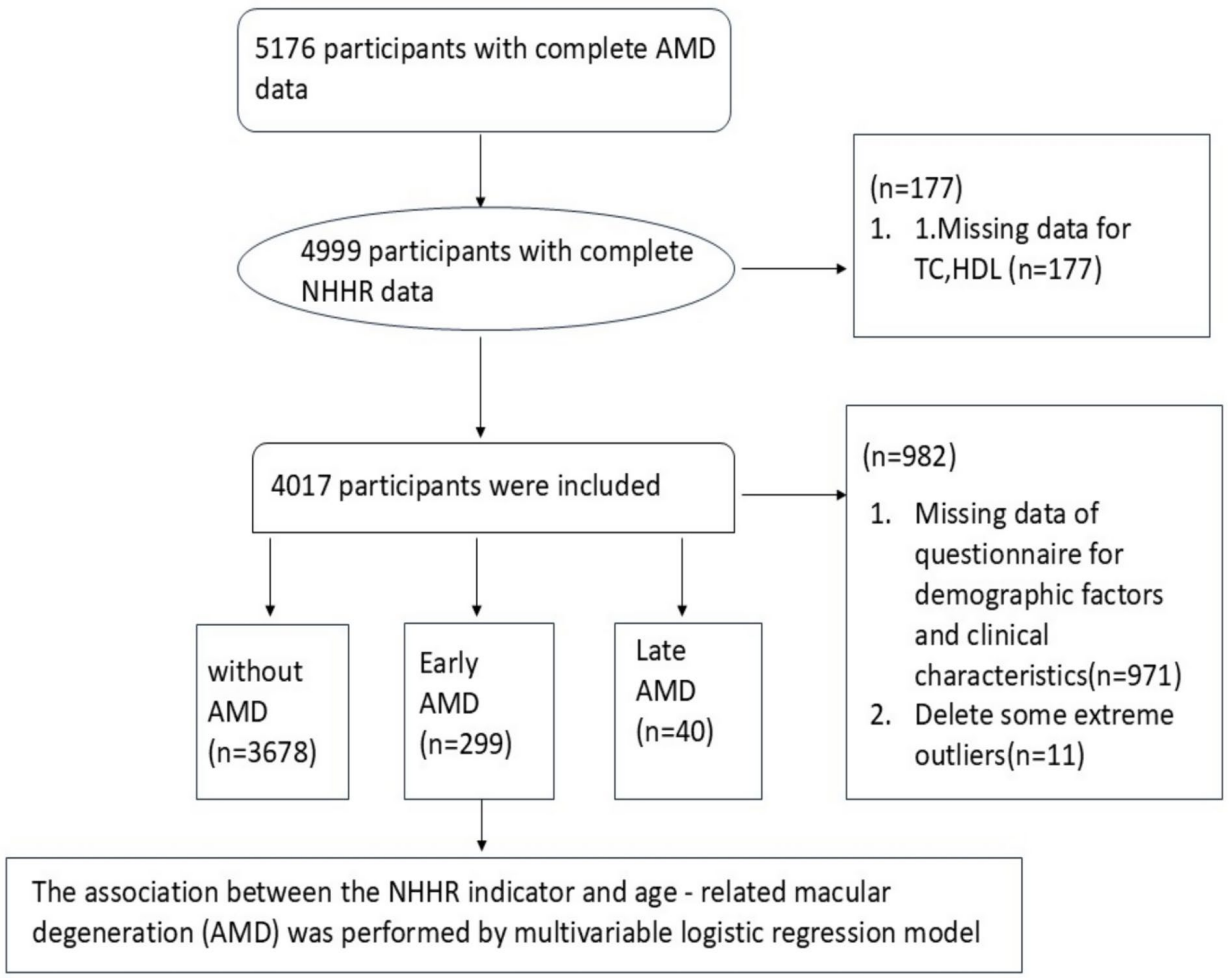
Flow chart of sample selection from NHANES 2005–2008.

From the 2005–2008 NHANES cycles, we included participants aged ≥40 years who did not meet any of the exclusion criteria and were eligible. The exclusion criteria included blindness (i.e., no light perception in both eyes), eye infections, or both eyes being covered by patches. There were a total of 5,176 individuals who met the criteria and had a complete AMD diagnosis. A total of 1,159 participants with missing data or outliers were excluded, as shown in Figure 1. Participants with missing data on any variable involved in the primary analysis were excluded, as the missingness was sporadic across variables and exhibited no specific pattern, consistent with being missing at random. Extreme outliers identified by the boxplot method were also removed. Ultimately, 4,017 participants (1,941 males and 2,076 females) were included in the study.

### 2.2 Diagnosis of AMD

Retinal images were acquired using a Canon CR6-45NM Ophthalmic Digital Imaging System with a Canon EOS 10D digital camera (Canon United States, Inc.). Participants were instructed to fixate on a target to ensure proper visual field alignment during image acquisition. When bilateral images were available, the eye exhibiting more advanced pathology was selected for analysis. Digital images were archived on DVDs and transferred to the University of Wisconsin-Madison for grading using the modified Wisconsin Age-Related Maculopathy Grading System (19). Early AMD was defined by the presence of drusen and/or pigment abnormalities, while late AMD is characterized by signs of exudative AMD and/or geographic atrophy. The primary outcome was AMD diagnosis (yes/no). Early/intermediate AMD and late AMD cases were combined for analysis. All images were independently graded by two certified evaluators, with a third senior grader resolving discrepancies.

### 2.3 Assessment of NHHR

NHHR is the ratio of serum non-HDL-C to HDL-C. The value of non-HDL-C is obtained by subtracting the HDL-C from the total cholesterol level (TC) of the subjects. Then, the obtained non-HDL-C level is divided by the HDL level to get the final NHHR data of the participants. The blood samples in this cross-sectional study were collected, processed, and stored in the laboratory of the Mobile Examination Center (MEC), and then transported to the Lipoprotein Analysis Laboratory of Johns Hopkins University for analysis and determination. Serum HDL-C levels were determined by direct immunochemistry, while total cholesterol was assayed by an enzymatic method.

### 2.4 Covariates

For this investigation in NHANES, potential confounding variables that might influence the association between NHHR and AMD were collected. The selected variables for analysis included: age (years), gender (male or female), ethnicity (categorized as Mexican American, other Hispanic, non-Hispanic White, non-Hispanic Black, or other race), marital status (categorized as never married, married or living with partner, and divorced, separated, or widowed), smoking status (determined by inquiring whether participants had ever smoked at least 100 cigarettes in their lifetime) (yes/no), hypertension (yes/no), drinking status (based on having had at least 12 alcoholic beverages in a lifetime) (yes/no), levels of TC, HDL-C, non-HDL-C (mg/dL), body mass index (BMI, kg/m^2^), high cholesterol status (yes/no), and diabetes status.

### 2.5 External validation cohort

To further verify the association between NHHR and the risk of AMD, we retrospectively collected data from participants at PHFT from November 2021 to September 2025. All participants meeting the study inclusion criteria were selected from the hospital-wide integrated informatics platform for clinical research. Then, a control group was established using a 1:1 random design. Ultimately, data from 48 AMD patients (disease group) and 48 non-AMD subjects (control group) were collected. The inclusion criteria were as follows: (1) age ≥40 years; (2) having a confirmed diagnosis of AMD or being confirmed as having no AMD by an ophthalmologist. The exclusion criteria were as follows: (1) participants with missing key study data; (2) participants whose serum marker testing time fell outside the concurrent study period; (3) participants with blindness (i.e., no light perception in both eyes), ocular infection, or plaque covering both eyes; (4) participants with other ocular diseases that affect image interpretation and interfere with study results (e.g., diabetic retinopathy, retinal artery and vein occlusion, high myopia-related macular degeneration, macular hole, retinal detachment, etc.). This protocol was approved by the Ethics Review Committee of PHFT. Since we only reviewed the existing database, the Ethics Committee waived the requirement of obtaining informed consent from patients.

### 2.6 Statistical analysis

Statistical analysis was conducted using EmpowerRCH (version 4.2) and R (version 4.4.2) (packages: survey, rms, forestplot, pROC, and ggplot2). In the cross-sectional study part based on the NHANES database, the statistical analysis was conducted following the procedures recommended by the Centers for Disease Control and Prevention (CDC). To account for the complex survey design and non-response rates observed in the NHANES, sample weights were employed in the analyses. In this study, half of the WTMEC2YR value was used as the weight for logistic regression analyses (20), while the stratification variable (STRATA) and the primary sampling unit variable (PSU) were incorporated to construct the survey design, correcting for biases introduced by the complex sampling scheme. The weights were calibrated to account for differences in sampling probabilities across sub-study populations and were consistent with the inclusion criteria of the sub-studies, ensuring that the analysis results are nationally representative. Continuous variables were presented as mean ± standard error (mean ± SE), while categorical variables were presented as numbers and weighted percentages (*n*, weighted %). Subsequently, a series of weighted logistic regression models was employed to explore the association between NHHR and AMD. Model 0 was an unadjusted model, serving as a baseline for comparison. Model 1 incorporated adjustments for confounding factors, such as gender, age, marital status, and ethnicity, aiming to control for the potential influence of these demographic variables. Model 2, built upon Model 1, further adjusted for lifestyle and health-related factors, specifically alcohol consumption, smoking status, BMI, hypertension, and diabetes mellitus, to more comprehensively account for the impact of various risk factors on the relationship under investigation. In addition, in the sensitivity analysis, we did not weight the data and adopted multivariable logistic regression analysis to further verify the stability of the results.

To investigate nonlinear relationships or dose–response connections, a weighted restricted cubic spline (RCS) was used. Subgroup analyses were performed using stratified multivariate regression models, stratified by age, gender, BMI, hypertension, and diabetes mellitus. Additionally, interaction tests were carried out to formally assess the heterogeneity of the associations among subgroups and applied false discovery rate (FDR) correction to the corresponding *p*-values using the Benjamini–Hochberg method. This correction step was taken to control for the bias potentially introduced by multiple testing. To assess the predictive performance of the models, discrimination was evaluated by receiver operating characteristic (ROC) curves and the area under the curve (AUC). Model calibration was assessed using calibration plots with bootstrapping that incorporated sampling weights, while the clinical utility was evaluated via decision curve analysis applied to the weighted cohort. Statistical significance was defined as a *p*-value less than 0.05, adhering to the conventional standard in scientific research for determining the reliability of observed associations.

In the part of the retrospective study based on the clinical data of PHFT, logistic regression was employed to validate the above conclusions. First, we performed a baseline analysis of the in-hospital data for the included patients with AMD and non-AMD individuals. Continuous variables were expressed as mean ± standard deviation (mean ± SD), while categorical variables were presented as numbers and percentages (*n*, %). Subsequently, a logistic model was used to explore the association between NHHR and AMD. Model 0 was an unadjusted model, serving as the baseline for comparison. Model 1 was a fully adjusted model, which adjusted for age and hypertension to account for the impact of various risk factors on the relationship under investigation. Thereafter, RCS analysis was conducted. ROC curve, AUC, calibration plot, and DCA were performed to evaluate model performance. Due to the small sample size of the in-hospital data, further subgroup splitting would lead to extremely low statistical power of the results, so subgroup analysis was not performed.

## 3 Results

### 3.1 Baseline characteristics of participants

In the 2005–2008 NHANES, a total of 5,176 participants underwent a comprehensive assessment and had complete diagnostic data for AMD. Subsequently, a rigorous data screening process was implemented. Finally, 4,017 people were included in the data analysis, including 1,941 (weighted 48.3%) males and 2,076 (weighted 51.7%) females. A total of 339 unweighted participants were diagnosed with AMD. The overall prevalence of AMD was 8.44% with a mean age of 67.55 ± 1.03 years. The majority of patients with AMD were men (*n* = 174; 46.94%). The most common ethnicity was non-Hispanic White, including 249 participants (weighted 88.11%) (Table 1).

**Table 1:** Baseline characteristics of adults in NHANES 2005–2008.

Variables	Total (*n* = 4,017)	Without AMD (*n* = 3,678)	AMD (*n* = 339)	*p*-value
Age, (mean ± SE)	56.66 (0.4)	55.86 (0.37)	67.55 (1.03)	<0.0001
Gender, *n* (weighted %)				0.6594
Male	1941 (48.3%)	1767 (45.73)	174 (46.94)	
Female	2076 (51.7%)	1911 (54.27)	165 (53.06)	
Ethnicity, *n* (weighted %)				0.0011
Mexican American	543 (13.5%)	507 (4.36)	36 (3.22)	
Other Hispanic	262 (6.5%)	245 (2.90)	17 (1.95)	
Non-Hispanic white	2,331 (58.0%)	2082 (79.87)	249 (88.11)	
Non-Hispanic Black	762 (19.0%)	732 (8.67)	30 (3.97)	
Other Race	119 (3.0%)	112 (4.21)	7 (2.75)	
Marital status, *n* (weighted %)				<0.0001
Never married	251 (6.2%)	239 (5.75)	12 (1.74)	
Married or living with partner	2,649 (65.9%)	2,456 (71.64)	193 (62.96)	
Divorced, separated, or widowed	1,117 (27.8%)	983 (22.60)	134 (35.31)	
Smoke, *n* (weighted %)				0.0113
Yes	2051 (51.1%)	1859 (48.76)	192 (58.80)	
Never	1966 (48.9%)	1819 (51.24)	147 (41.20)	
Alcohol, *n* (weighted %)				0.0807
Yes	2,757 (68.6%)	2,535 (73.28)	222 (67.50)	
No	1,260 (31.4%)	1,143 (26.72)	117 (32.50)	
Hyperlipidemia, *n* (weighted %)				0.5310
Yes	2057 (51.2%)	1881 (48.92)	176 (50.86)	
No	1960 (48.8%)	1797 (51.08)	163 (49.14)	
BMI (kg/m^2^), mean ± SE	29.28 (0.17)	28.91 (0.17)	30.10 (0.42)	0.3824
Hypertension, *n* (weighted %)				0.0033
Yes	1969 (49.0%)	1773 (43.18)	196 (54.16)	
No	2048 (51.0%)	1905 (56.82)	143 (45.84)	
DM, *n* (weighted %)				0.3343
Yes	3,258 (81.1%)	2,983 (86.85)	275 (85.03)	
No	759 (18.9%)	695 (13.15)	64 (14.97)	
NHHR (mean ± SE)	3.03 (0.03)	3.05 (0.03)	2.83 (0.07)	0.0094
NHHR tertile				0.0597
Low	33.43 (0.77)	33.04 (0.81)	33.80 (3.02)	
Middle	33.29 (0.87)	33.19 (0.91)	34.67 (3.30)	
High	33.27 (0.79)	33.77 (0.85)	26.53 (2.79)	
TC, (mg/dL), mean ± SE	203.11 (0.84)	203.30 (0.89)	200.60 (2.96)	0.3942
HDL-C, (mg/dL), mean ± SE	54.08 (0.35)	53.9 (0.362)	56.5 (0.96)	0.012
Non-HDL-C, (mg/dL), mean ± SE	149.03 (0.82)	149.4 (0.86)	144.1 (2.88)	0.091

Continuous variables were presented as survey-weighted mean (95% CI). The *p*-value was calculated by a survey-weighted logistic regression model. Categorical variables were presented as survey-weighted percentages (95% CI). The *p*-value was calculated by a survey-weighted chi-squared test. AMD, age-related macular degeneration; BMI, body mass index; DM, diabetes mellitus; NHHR, the non-high-density lipoprotein cholesterol to high-density lipoprotein cholesterol ratio; TC, total cholesterol; non-HDL-C, non-high-density lipoprotein cholesterol; HDL-C, high-density lipoprotein cholesterol.

There were significant differences between participants with and without AMD in age, ethnicity, marital status, smoke, hypertension, HDL-C, and NHHR (*p* < 0.05). However, when the NHHR index was categorized into tertiles, the difference became less evident under this classification (*p* = 0.0597). It indicates that within this classification framework, the relationship between NHHR and AMD may not follow a simple categorical pattern or may be interfered with by potential confounding factors.

The data of study participants from the People’s Hospital Affiliated to Fujian University of Traditional Chinese Medicine were complete and required no processing. In this study, there were a total of 96 participants. Among the 48 recruited AMD patients, the mean age was 75.54 years, 41.67% of the participants were female, and the majority of AMD patients were male (*n* = 28; 58.33%) (Table 2). Significant differences were observed between subjects with and without AMD in terms of age, hypertension, NHHR, and non-HDL-C (*p* < 0.05).

**Table 2:** Baseline characteristics of adults in PHFT.

Variables	Total (*n* = 96)	Without AMD (*n* = 48)	AMD (*n* = 48)	*p*-value
Age (mean ± SD)	70.76 (11.53)	65.98 (11.8)	75.54 (9.22)	<0.001
Gender, *n* (weighted %)				0.102
Male	48 (50%)	20 (41.67)	28 (58.33)	
Female	48 (50%)	28 (58.33)	20 (41.67)	
BMI (kg/m^2^), mean ± SD	24.2 (2.99)	24.56 (3.04)	23.84 (2.92)	0.335
Hypertension, *n* (weighted %)				<0.001
Yes	56 (58.33%)	18 (37.5)	30 (79.17)	
No	40 (41.67%)	30 (62.5)	18 (20.83)	
DM, *n* (weighted %)				0.210
Yes	38 (39.58%)	22 (13.15)	16 (14.97)	
No	58 (81.1%)	26 (86.85)	32 (85.03)	
NHHR (mean ± SE)	3.51 (1.34)	3.91 (1.26)	3.12 (1.31)	0.003
TC, (mmol/L), mean ± SD	4.95 (1.12)	5.08 (1.03)	4.82 (1.20)	0.3942
Non-HDL-C (mmol/L), mean ± SD	3.77 (1.01)	4.01 (0.94)	3.54 (1.04)	0.023
HDL-C (mmol/L), mean ± SD	1.16 (0.38)	1.07 (0.24)	1.26 (0.46)	0.061

Continuous variables were presented as survey mean (95% CI). The *p*-value was calculated by a survey logistic regression model. Categorical variables were presented as survey percentages (95% CI). The *p*-value was calculated by a survey chi-squared test. AMD, age-related macular degeneration; BMI, body mass index; DM, diabetes mellitus; NHHR, the non-high-density lipoprotein cholesterol to high-density lipoprotein cholesterol ratio; TC, total cholesterol; non-HDL-C, non-high-density lipoprotein cholesterol; HDL-C, high-density lipoprotein cholesterol.

### 3.2 Association between NHHR and AMD

In NHANES, the unadjusted model demonstrated a statistically significant inverse correlation between NHHR and AMD (OR = 0.873, 95% CI: 0.79–0.97, *p* = 0.014). This association remained significant in Model 1 (adjusted for basic demographic variables) and the fully adjusted Model 2 (Model 1: OR = 0.879, 95% CI: 0.79–0.98, *p* = 0.023; Model 2: OR = 0.868, 95% CI: 0.78–0.97, *p* = 0.016). In the analyses based on NHHR tertiles, all three models exhibited significant negative associations in the highest NHHR tertile (Model 0: OR = 0.669, 95% CI: 0.48–0.93, *p* = 0.018; Model 1: OR = 0.682, 95% CI: 0.48–0.96, *p* = 0.023; Model 2: OR = 0.669, 95% CI: 0.47–0.95, *p* = 0.025). The results of the trend analyses indicated the presence of a significant dose–response relationship in three models (*p* for trend = 0.022, 0.028, and 0.023, respectively; Table 3). In the PHFT data of unadjusted analyses, NHHR showed statistically significant inverse associations with AMD (OR = 0.596, 95% CI: 0.413–0.861, *p* = 0.006). In the analysis based on NHHR tertiles, Model I showed a significant negative correlation in the highest NHHR tertile (Model 0: OR = 0.238, 95% CI: 0.084–0.677, *p* = 0.007; Model 1: OR = 0.232, 95% CI: 0.072–0.750, *p* = 0.015). The results of trend analysis indicated that there was a significant dose–response relationship in both models (*p* for trend = 0.008 and 0.015, respectively) (Table 4).

**Table 3:** Associations between NHHR and its tertile and AMD among participants (NHANES).

Variables	Non-adjusted OR (95% CI)	*p*-value	Adjust I OR (95% CI)	*p*-value	Adjust II OR (95% CI)	*p*-value
NHHR	0.873 (0.79, 0.97)	0.014	0.879 (0.79, 0.98)	0.023	0.868 (0.78, 0.97)	0.016
NHHR tertile						
Q1	1.0 (ref.)		1.0 (ref.)		1.0 (ref.)	
Q2	0.889 (0.64, 1.24)	0.161	0.880 (0.63, 1.24)	0.448	0.89 (0.63, 1.26)	0.506
Q3	0.669 (0.48, 0.93)	0.018	0.682 (0.48, 0.96)	0.023	0.669 (0.47, 0.95)	0.025
*p* for trend	0.85 (0.74, 0.98)	0.022	0.83 (0.70, 0.98)	0.028	0.821 (0.69, 0.97)	0.023

Model 0: no covariates were adjusted; Model 1: gender, age, and race were adjusted; Model 2: gender, age, race, marital status, BMI, smoking status, drinking status, diabetes, and hypertension were adjusted. AMD, age-related macular degeneration; BMI, body mass index; NHHR, the non-high-density lipoprotein cholesterol to high-density lipoprotein cholesterol ratio; OR, odds ratio; 95% CI, confidence interval.

**Table 4:** Associations between NHHR and its tertile and AMD among participants (PHFT).

Variables	Non-adjusted OR (95% CI)	*p*-value	Adjust I OR (95%CI)	*p*-value
NHHR	0.596 (0.413, 0.861)	0.006	0.592 (0.400, 0.874)	0.008
NHHR tertile				
Q1	1.0 (ref.)		1.0 (ref.)	
Q2	0.401 (0.145, 1.112)	0.079	0.451 (0.144, 1.411)	0.171
Q3	0.238 (0.084, 0.677)	0.007	0.232 (0.072, 0.750)	0.015
*p* for trend	0.535 (0.337, 0.847)	0.008	0.527 (0.314, 0.883)	0.015

Model 0: no covariates were adjusted; Model 1: age and hypertension were adjusted. AMD, age-related macular degeneration; NHHR, the non-high-density lipoprotein cholesterol to high-density lipoprotein cholesterol ratio; OR, odds ratio; 95% CI, confidence interval.

Based on parallel models constructed to compare the associations of single lipid indicators with AMD, NHHR demonstrated consistently significant associations across all model specifications in both the NHANES and external validation cohorts. In contrast, non-HDL-C and HDL-C showed significant associations only in selected models or specific subgroup analyses. These findings suggest that NHHR is a more robust and consistent predictor of AMD than individual lipid measures (Supplementary Tables S1–S4).

### 3.3 Restricted cubic spline model of NHHR and AMD

**Figure 2:**
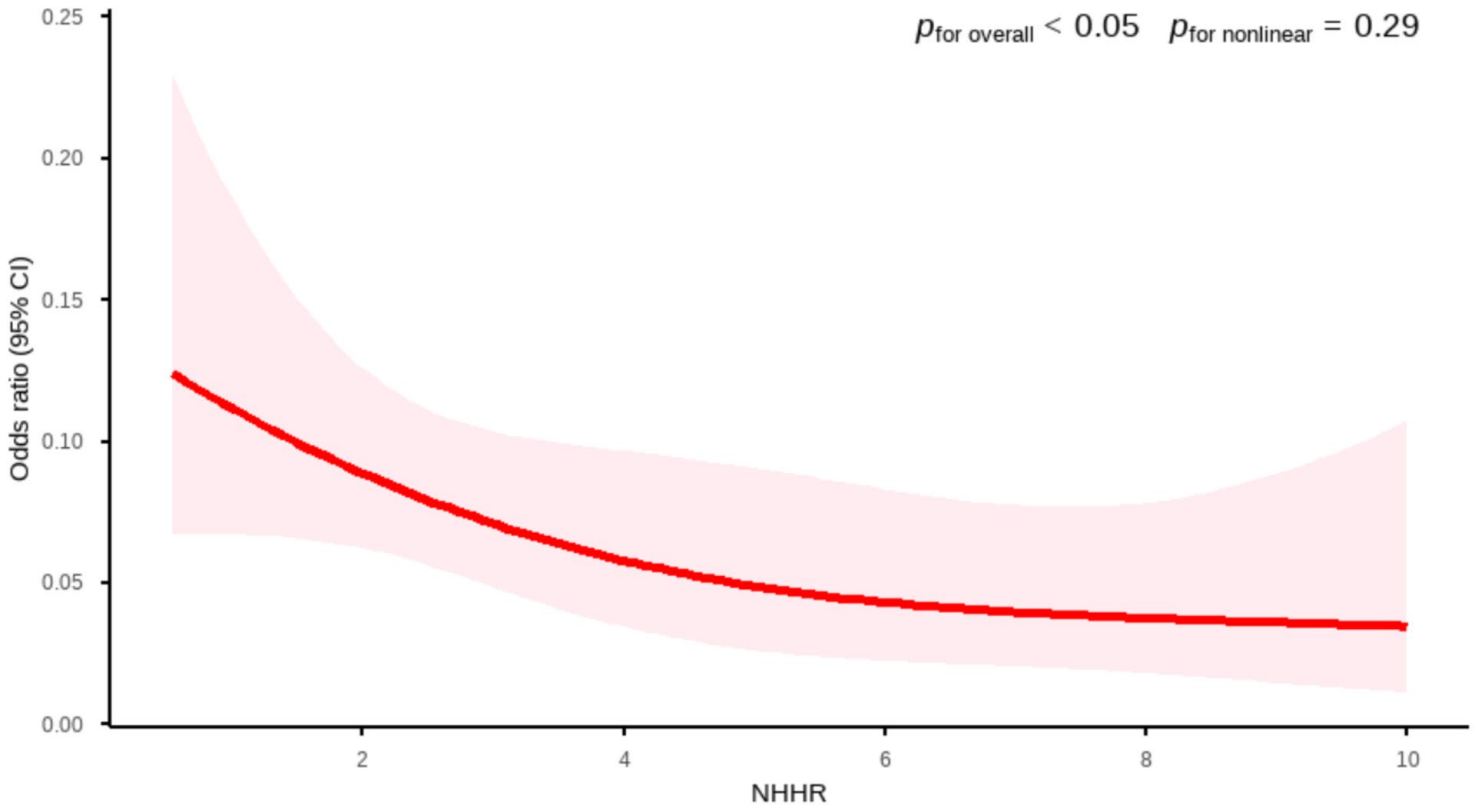
Association between NHHR and AMD by RCS curve (NHANES). Age, gender, race, marital status, BMI, smoking status, drinking status, diabetes, and hypertension were adjusted.

To investigate the potential non-linear association between NHHR and AMD, this study employed a weighted RCS model for fitting analysis. In the large-sample NHANES dataset, RCS analysis revealed a statistically significant inverse relationship between NHHR and AMD (*p* for overall <0.05). The association curve exhibited an approximately linear trend (*p* for nonlinear = 0.29). This finding indicates that in the primary dataset, the association between NHHR and AMD is more consistent with a linear relationship (Figure 2).

**Figure 3:**
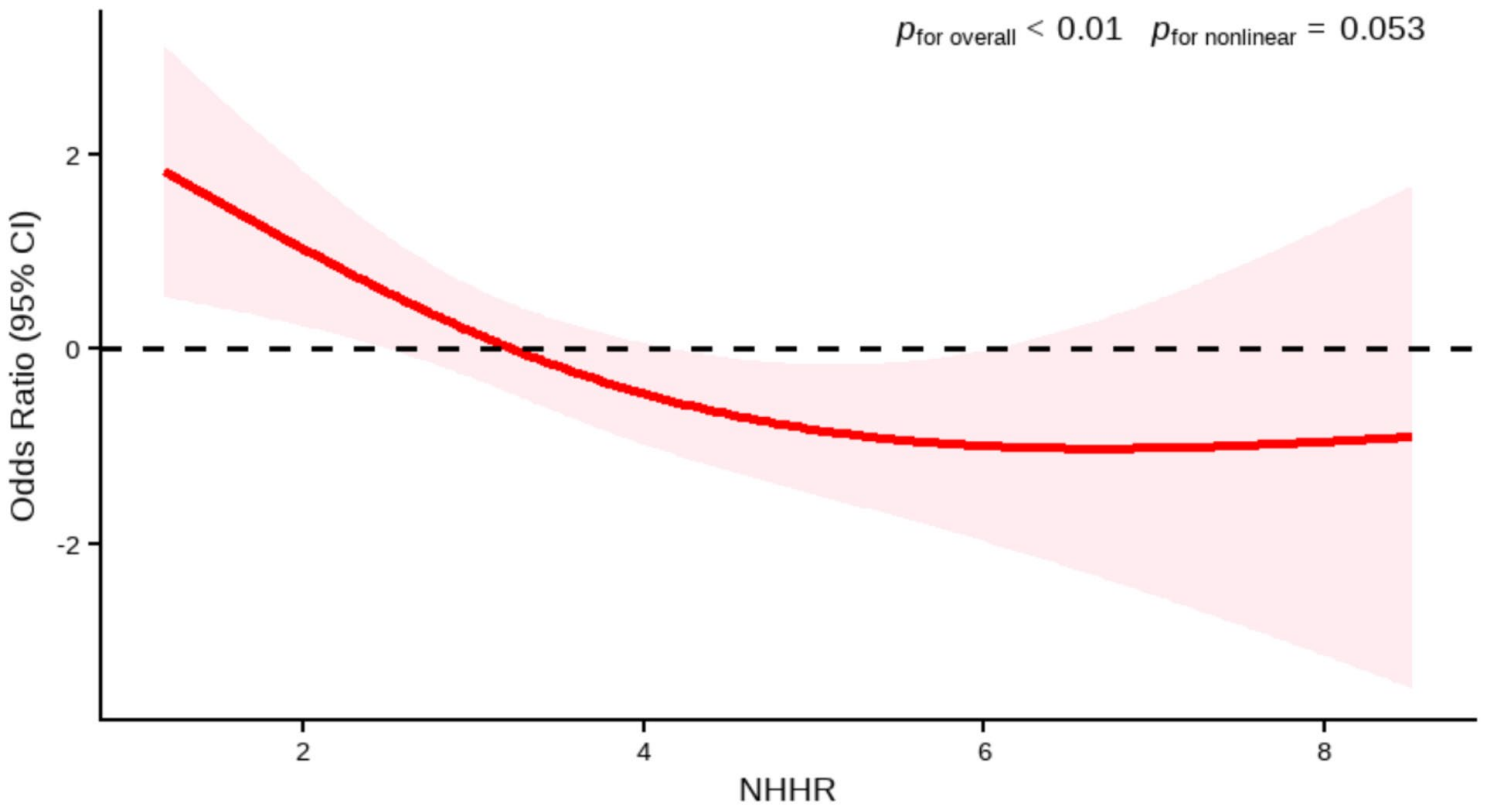
Association between NHHR and AMD by RCS (PHFT). Age, hypertension were adjusted.

In the external PHFT validation set (*n* = 96), the RCS model also confirmed a significant inverse relationship between the two variables (*p* for overall <0.01). Although the shape of the RCS curve suggested a potential non-linear association trend, the non-linearity test did not reach the conventional threshold for statistical significance (*p* for nonlinear = 0.053). Given the limited sample size and consequent low statistical power in this validation set, the current results are insufficient to confirm the existence of a true non-linear relationship. Therefore, based on the current data, we failed to confirm the existence of a significant non-linear association. The relationship can be considered approximately linear, and a linear model was used for interpretation (Figure 3).

### 3.4 Subgroup analysis

**Figure 4:**
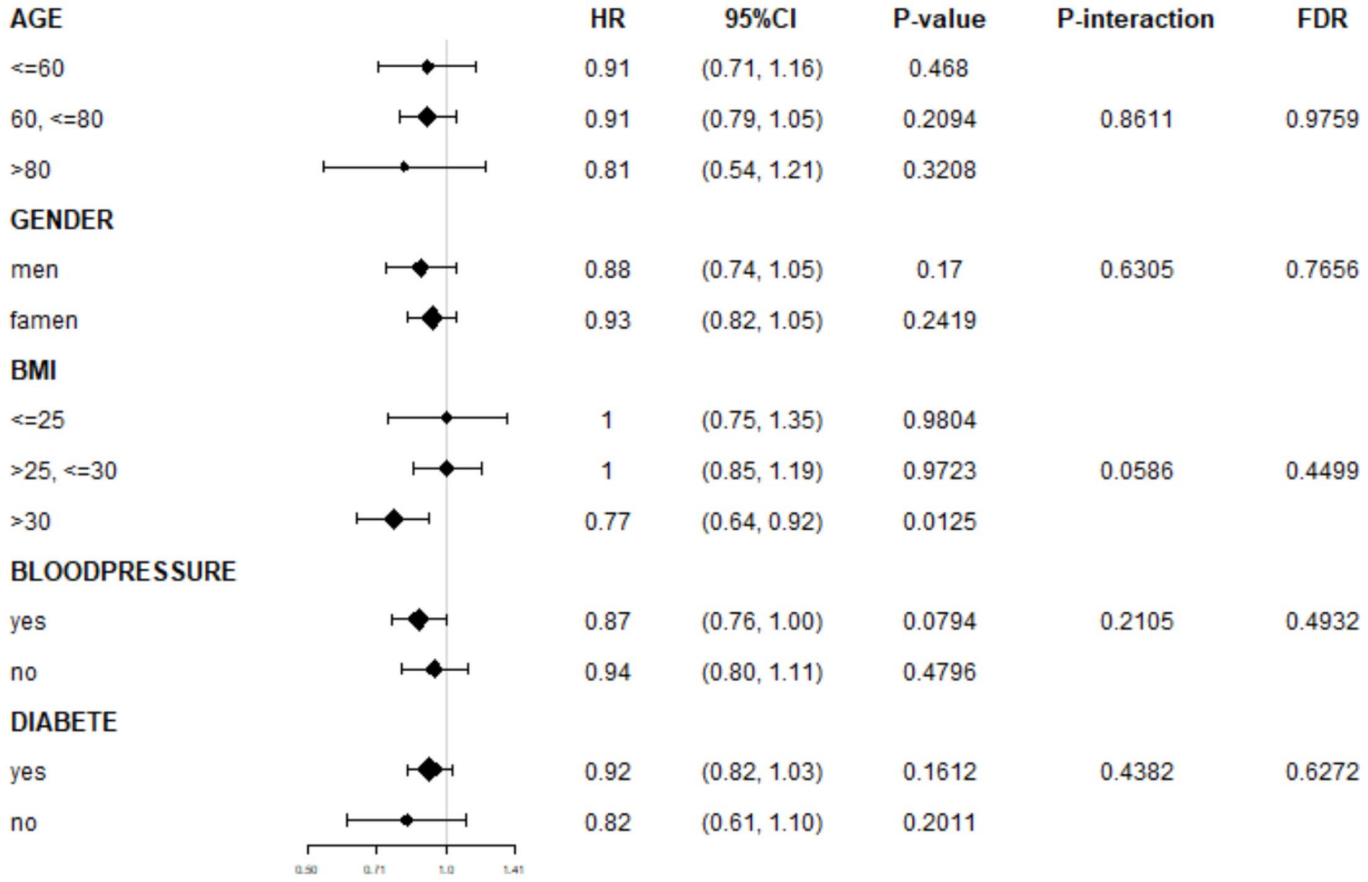
Subgroup analyses of association between NHHR and AMD.

We further assessed the consistency of the association between NHHR and AMD across different population characteristics through subgroup analyses. The results suggested a generally consistent direction of the association between NHHR and AMD across predefined subgroups, such as age, sex, hypertension, and diabetes. In the BMI subgroup analysis, an initial assessment revealed a statistically significant association between BMI >30 kg/m^2^ and AMD (OR: 0.77, 95% CI: 0.64–0.92, *p* = 0.0125). However, this association did not reach statistical significance after FDR correction (FDR-adjusted *p* = 0.2125), indicating that this result requires cautious interpretation (Figure 4). Moreover, after applying false discovery rate (FDR) correction to control for the risk of false positives from multiple comparisons, neither the association effects within subgroups nor the interaction terms reached statistical significance (all FDR-corrected *p* > 0.05; Supplementary material: FDR_Results).

The interaction analysis results further supported these findings. No significant differences in the strength of the association between NHHR and AMD were observed among participants stratified by age, sex, BMI category, hypertension, or diabetes status (all FDR-corrected *p* for interaction terms >0.05).

In summary, both the subgroup and interaction analyses indicate that the negative correlation between NHHR and AMD demonstrates relative consistency across demographic and clinical characteristic subgroups, with no significant effect modifiers identified.

### 3.5 Sensitivity analysis

According to the sensitivity analysis using unweighted logistic regression, in the fully adjusted model, NHHR remained negatively associated with AMD. Similarly, the NHHR tertile analysis and trend test for AMD were also statistically significant (Table 5). These results indicate that our findings are stable and robust.

**Table 5:** Unweighted logistic regression analysis of the correlation between NHHR and AMD.

Variables	Non-adjusted OR (95% CI)	*p*-value	Adjust I OR (95% CI)	*p*-value	Adjust II OR (95% CI)	*p*-value
NHHR	0.88 (0.81, 0.96)	0.0061	0.91 (0.83, 1.00)	0.0544	0.90 (0.82, 1.00)	0.0407
NHHR tertile						
Q1	1.0 (ref.)		1.0 (ref.)		1.0 (ref.)	
Q2	0.96 (0.74, 1.25)	0.7839	0.97 (0.74, 1.27)	0.7673	0.97 (0.74, 1.28)	0.8477
Q3	0.71 (0.54, 0.94)	0.0181	0.73 (0.54, 0.97)	0.0321	0.73 (0.54, 0.98)	0.0375
*p* for trend	0.87 (0.78, 0.97)	0.0149	0.88 (0.78, 0.98)	0.0263	0.88 (0.78, 0.99)	0.0302

### 3.6 Model performance and clinical utility

**Figure 5:**
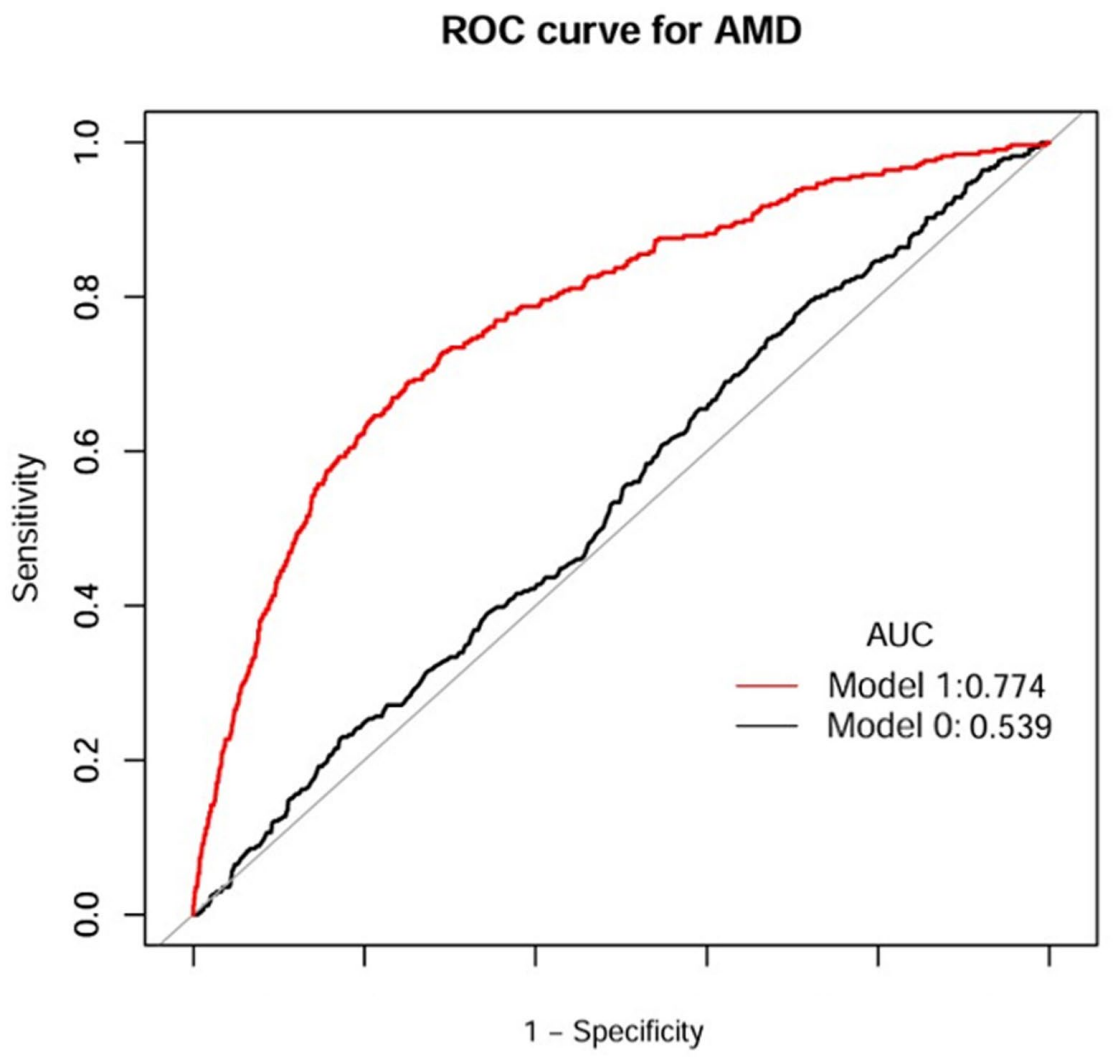
ROC curves of NHHR index for the prediction of AMD (NHANES). Model 1 was Model 0 plus gender, age, race, marital status, BMI, smoking status, drinking status, diabetes, and hypertension; Model 0 included NHHR. ROC, receiver operating characteristic curve; AUC, area under curve; AMD, age-related macular degeneration; BMI, body mass index; NHHR, the non-high-density lipoprotein cholesterol to high-density lipoprotein cholesterol ratio.

**Figure 6:**
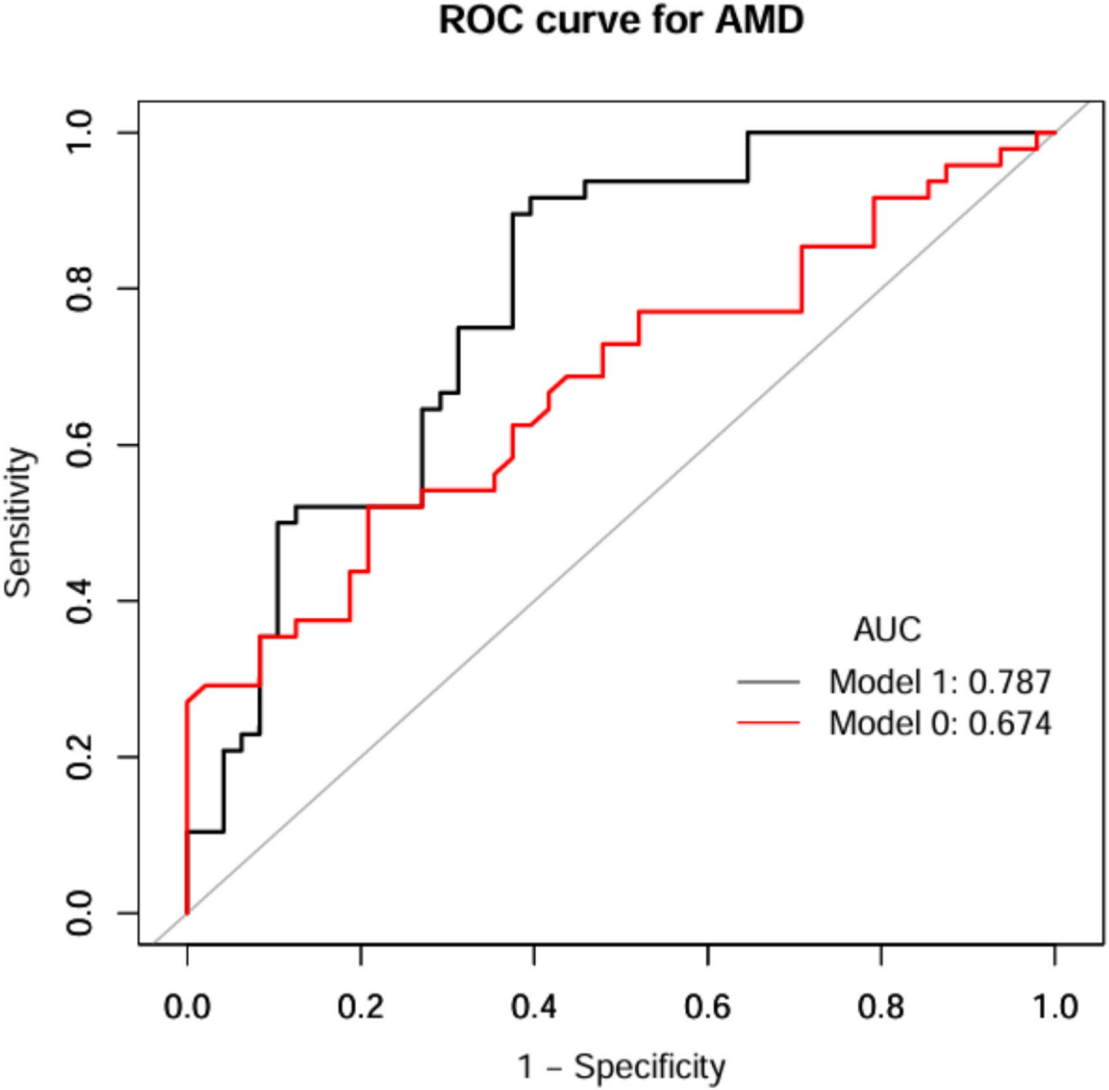
ROC curves of NHHR index for prediction of AMD (PHFT). Model 1 was Model 0 plus age and hypertension; Model 0 included NHHR. ROC, receiver operating characteristic curve; AUC, area under curve; AMD, age-related macular degeneration; NHHR, the non-high-density lipoprotein cholesterol to high-density lipoprotein cholesterol ratio.

The discriminative performance of the model incorporating NHHR was assessed using receiver operating characteristic (ROC) analysis and quantified by the area under the curve (AUC). In the NHANES dataset, the fully adjusted model (Model I) achieved an AUC of 0.774 (95% CI: 0.75–0.80) for predicting AMD, demonstrating a significant improvement in predictive performance compared to the initial model (Model 0: AUC = 0.539, 95% CI: 0.51–0.57) (Figure 5 and Table 6). Consistent results were obtained from external validation in the PHFT cohort (Model I: AUC = 0.787, 95% CI: 0.69–0.88; Model 0: AUC = 0.674, 95% CI: 0.57–0.78) (Figure 6 and Table 7). To further validate the unique predictive value of the NHHR index, we additionally compared NHHR with its individual components. The results indicated that the NHHR index outperformed the use of either non-HDL-C or HDL-C alone in distinguishing AMD cases (Supplementary Figures S1, S2 and Supplementary Tables S5, S6). The calibration curve indicates that the model has good calibration performance (Supplementary Figures S3, S4). Hosmer–Lemeshow test results indicated no statistically significant difference between predicted risks and actual AMD prevalence rates (NHANES: *χ*^2^ = 14.95, *p* = 0.062; PHFT: *χ*^2^ = 4.17, *p* = 0.24). Compared with the traditional risk-based baseline model, the incorporation of NHHR has yielded a significant improvement in model performance across both internal and external studies (NHANES: NRI = 0.84, IDI = 0.09, both *p* < 0.01; PHFT: NRI = 0.83, IDI = 0.22, both *p* < 0.010).

**Table 6:** Diagnostic efficacy of ROC analysis of anthropometric indices for AMD (NHANES).

Test	Best threshold	Accuracy	Sensitivity	Specificity	Positive predictive value	Negative predictive value	AUC (95% CI)
Model 0	3.5850	0.3368	0.7788	0.2961	0.0925	0.9356	0.539 (0.51, 0.57)
Model 1	−2.1879	0.7461	0.6873	0.7515	0.2031	0.9631	0.774 (0.75, 0.80)

ROC, receiver operating characteristic curve; AMD, age-related macular degeneration; NHHR, the non-high-density lipoprotein cholesterol to high-density lipoprotein cholesterol ratio; AUC, area under the curve; 95% CI, confidence interval.

**Table 7:** Diagnostic efficacy of ROC analysis of anthropometric indices for AMD (PHFT).

Test	Best threshold	Accuracy	Sensitivity	Specificity	Positive predictive value	Negative predictive value	AUC (95% CI)
Model 0	3.0350	0.6562	0.5208	0.7971	0.7143	0.6230	0.674 (0.57, 0.78)
Model 1	0.4829	0.7604	0.8958	0.6250	0.7049	0.8571	0.787 (0.69, 0.88)

ROC, receiver operating characteristic curve; AMD, age-related macular degeneration; NHHR, the non-high-density lipoprotein cholesterol to high-density lipoprotein cholesterol ratio; AUC, area under the curve; 95% CI, confidence interval.

Using DCA, we evaluated the clinical utility of the NHHR-enhanced model across different populations. Based on the NHANES database, which showed that despite the low prevalence of AMD in the study population (consistent with general epidemiological characteristics), the model still demonstrated clinical reference value at lower decision thresholds (approximately 15%). However, in the external validation cohort, the model exhibited stronger generalization capability: across a wide range of clinically reasonable threshold probabilities (20–80%), its net benefit was superior to both the “treat all” and “treat none” strategies. This comparative result suggests that the NHHR model has significant clinical application potential in target populations with a higher prevalence of AMD, enabling the optimization of intervention strategies and improvement of patient outcomes through accurate identification of high-risk individuals (Supplementary Figures S5, S6).

## 4 Discussion

This study first reveals that the NHHR index has a negative relationship with the development of AMD. The part of cross-sectional analysis based on NHANES reveals the NHHR index exhibited a significant linear negative correlation with AMD. The other part of the retrospective study from PHFT also confirmed the linear negative correlation between the NHHR index and AMD.

NHHR is a ratio calculated from non-HDL-C and HDL-C, and this index is closely associated with blood lipid levels. Emerging as a novel biomarker, it has demonstrated clinical relevance across multiple systemic diseases, such as depression, hypertension, diabetes mellitus, and nephrolithiasis (14, 21–23). Although no previous studies have reported the relationship between the NHHR index and the prevalence of AMD, there is a great deal of literature on the association between lipid parameters related to NHHR and the disease. Fan et al. (24) found in their research that high levels of plasma HDL-C are causally related to an increased risk of advanced AMD in European and Asian populations. Burgess et al. (25) applied Mendelian randomization studies to confirm that high HDL-C is a pathogenic risk factor for AMD. Shen et al. (26) revealed that an increase in circulating HDL-C levels raises the risk of early AMD through MR analysis. Theoretically, HDL-C has anti-inflammatory, antioxidant functions, and promotes reverse cholesterol transport. So, as far as AMD is concerned, it should be regarded as a positive protective factor. However, Wang et al. (27) confirmed that the development of at least 40% of drusen is closely related to lipoproteins, indicating that high levels of HDL-C may promote the occurrence of drusen. Existing studies have confirmed that cholesterol and apolipoprotein B (ApoB) are stably present in drusen and basal deposits of both AMD patients and normal individuals (28). Another targeted study further revealed that with aging, neutral lipids gradually accumulate in Bruch’s membrane, and experiments have verified that these lipid deposits originate, at least in part, from lipoproteins produced by RPE cells. The mechanism underlying this accumulation is highly similar to the lipid deposition process in atherosclerosis (29). Moreover, the complement system plays a crucial role in the pathogenesis of AMD. HDL-C is correlated with multiple inflammatory mediators in the complement system. Genetically, AMD risk is associated with multiple HDL-C metabolic pathway genes. It includes the adenosine triphosphate-binding cassette transporter A1 (ABCA1), cholesteryl ester transfer protein (CETP), apolipoprotein E (APOE), and lipoprotein lipase C (LIPC) genes (30). Therefore, HDL-C cannot be considered a protective factor for AMD. It should not be overlooked that there are still a small number of studies presenting different viewpoints. For example, the research by Tan et al. (31) indicated that the development of advanced AMD is negatively correlated with an increase in HDL-C. However, looking at the current research findings as a whole, most reports show that HDL-C levels are positively correlated with an increased risk of AMD. While conflicting evidence exists, most studies indicate a positive HDL-C-AMD relationship. A newly published study puts forward for the first time that there is a U-shaped relationship between HDL-C and the risk of AMD, that is, both low and high levels of HDL-C can increase the risk of developing AMD (32). Therefore, the relationship between the HDL-C index and the prevalence of AMD remains controversial.

Non-HDL-C is the sum of all types of lipoprotein cholesterol in the blood except HDL, including LDL-C, very-low-density lipoprotein cholesterol (VLDL-C), intermediate-density lipoprotein cholesterol (IDL-C), etc. Research on the association between non-HDL-C and ophthalmic diseases is scarce. There are some studies on the relationship between single indicators and the risk of AMD. Wang et al. (33) found through a meta-analysis that LDL-C exhibits a protective effect in the development process of AMD. This conclusion stands in sharp contrast to the traditional perception that LDL-C is a risk factor for atherosclerosis, and this discrepancy may be attributed to the differences in the sources of lipoproteins (29). Nordestgaard et al. (34) discovered that an increase in HDL-C and a decrease in LDL-C were closely related to an increased risk of AMD in 106,703 individuals in the Canadian Genome and Population Study (CGPS). This seems to be consistent with the results of our study. Han et al. (35) demonstrated in their study through genetic evidence that non-HDL-C levels are specifically associated with a reduced risk of intermediate AMD and GA. This result can be partly attributed to the high heterogeneity of AMD in these studies, including different stages and subtypes. High levels of non-HDL-C usually indicate lipid metabolism disorders, and it may serve as a risk factor for diabetic retinopathy and retinal artery occlusion (36, 37). In conclusion, there are still a large number of unknown areas regarding the true relationship between non-HDL-C and AMD, as well as the underlying molecular mechanisms. Further research is warranted to elucidate these mechanisms.

Based on the above-mentioned situation, it is necessary for us to study the relationship between NHHR and AMD. By analyzing the data of participants from two settings, namely the NHANES database and PHFT data, we concluded that, compared with the participants without AMD, there is a significant negative correlation between the subjects with AMD and NHHR. To explore the generalizability of this study’s findings across populations with different characteristics, we conducted subgroup and interaction analyses using data from NHANES. The results show consistent associations between NHHR and AMD across various subgroups. This study also confirms that NHHR is a potentially useful tool for risk stratification in clinical practice.

Currently, the relationships between the HDL-C and non-HDL-C indicators and the prevalence of AMD remain controversial. The reasons for the differing results may lie in the differences in AMD classification, the impact of systemic and local lipid metabolism on lipid levels, and objective factors such as genetic or other factors that affect lipid levels. In summary, the exact mechanism underlying the currently recorded negative correlation trend between the NHHR indicator and the risk of AMD remains unclear. Future studies should attempt to overcome the limitations of the research and elaborate on the underlying mechanisms in detail.

## 5 Advantages and limitations

To the best of our knowledge, this is the first study to explore the association between NHHR and AMD by formulating a hypothesis based on cross-sectional data from the U.S. population and validating it with retrospective data from a tertiary hospital in China. Second, this study utilized the NHANES database, which has a large number of nationally representative samples, enhancing the reliability of the results. Furthermore, external validation data from China were used to verify the cross-regional and cross-ethnic generalizability of the study findings.

However, this article also has limitations. First, due to the cross-sectional study design employed in the database research of this article, the causal relationship between NHHR and AMD cannot be confirmed. The results of this study should be interpreted with caution, and future studies with larger sample sizes are needed to conduct longitudinal research. Second, the retrospective data from the hospital were all derived from “patients treated at a single center,” which may be subject to admission bias and selection bias. Additionally, the small sample size precluded accurate validation of the findings. Furthermore, even though we considered several potential covariates, the influence of other unmeasured confounding factors cannot be completely ruled out. Finally, due to the limitations of the investigable scope of AMD, we had to use the data from 2005–2008 in NHANES. However, this is the only AMD-related data in the current database, and the disease generally has a slow progression. Therefore, it still has a certain reference value for clinical practice. It is recommended that subsequent studies use updated and detailed clinical data, increase the sample size, and conduct multicenter prospective cohort studies to verify the robustness of the conclusions.

## 6 Conclusion

Our preliminary study shows that as the NHHR index increases, participants have a reduced likelihood of developing AMD. The inconsistent results with traditional studies can be partly explained by factors such as high HDL-C levels promoting drusen formation, excessive activation of inflammatory complement, and genetic metabolic pathways, while relatively low non-HDL-C levels are associated with differences in lipoprotein sources and the high heterogeneity of different stages and subtypes of AMD. This study provides insights for identifying novel serum biomarkers for AMD, and NHHR may be a reliable independent indicator for predicting the risk of AMD. In the future, large-scale sample studies and more prospective research are still needed to confirm our findings.

## Data Availability

The datasets presented in this study can be found in online repositories. The names of the repository/repositories and accession number(s) can be found below: https://www.cdc.gov/nchs/nhanes/.
