# Supplementary material for: Association between non-high-density lipoprotein cholesterol to high-density lipoprotein cholesterol ratio and age-related macular degeneration: insights from two observational studies
Source: Front Med (Lausanne). 2025 Dec 10;12:1724938. doi: 10.3389/fmed.2025.1724938 (PMC12728024; doi:10.3389/fmed.2025.1724938)
Supplement: Supplementary file 1 [file Data_Sheet_1.docx]

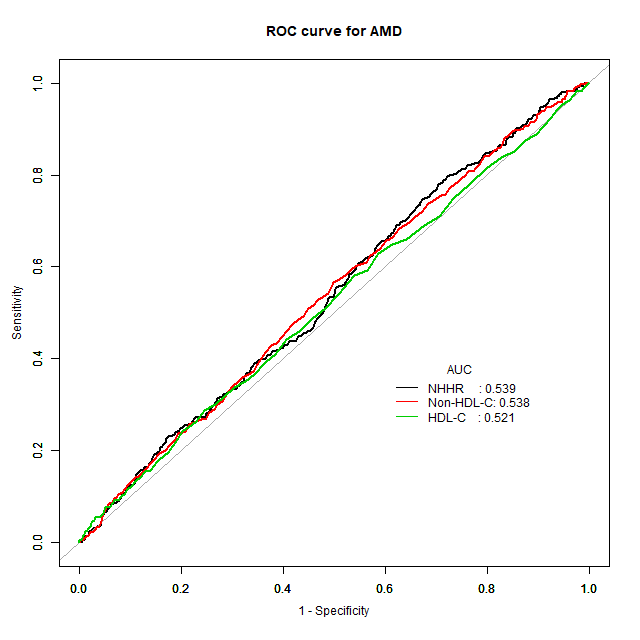


**Figure S1** Comparison of discriminative ability between NHHR and its component lipids for incident AMD(NHANES)

**
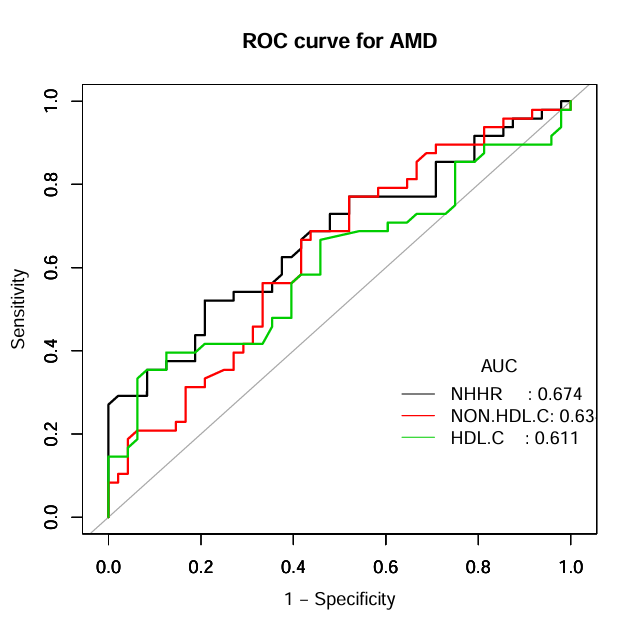
**

**Figure S2** Comparison of discriminative ability between NHHR and its component lipids for incident AMD(PHFT)

**
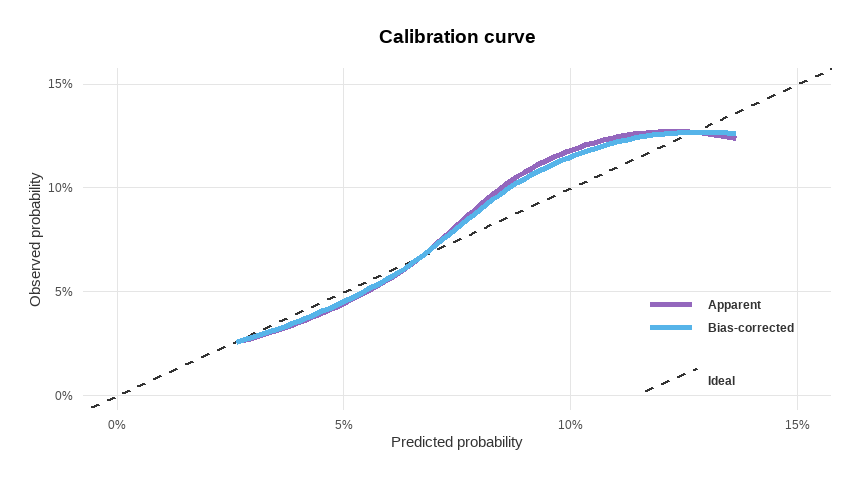
**

**Figure S3** Calibration Curve for AMD Risk Prediction by NHHR (NHANES)

**
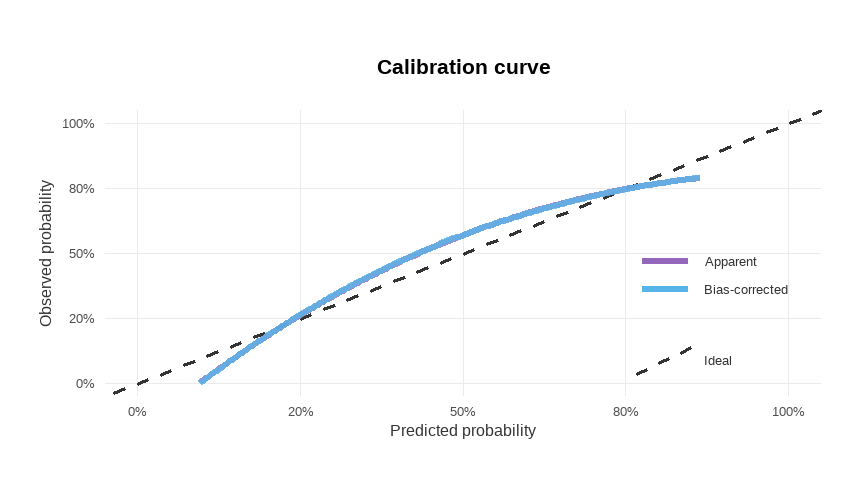
**

**Figure S4** Calibration Curve for AMD Risk Prediction by NHHR (PHFT)

**
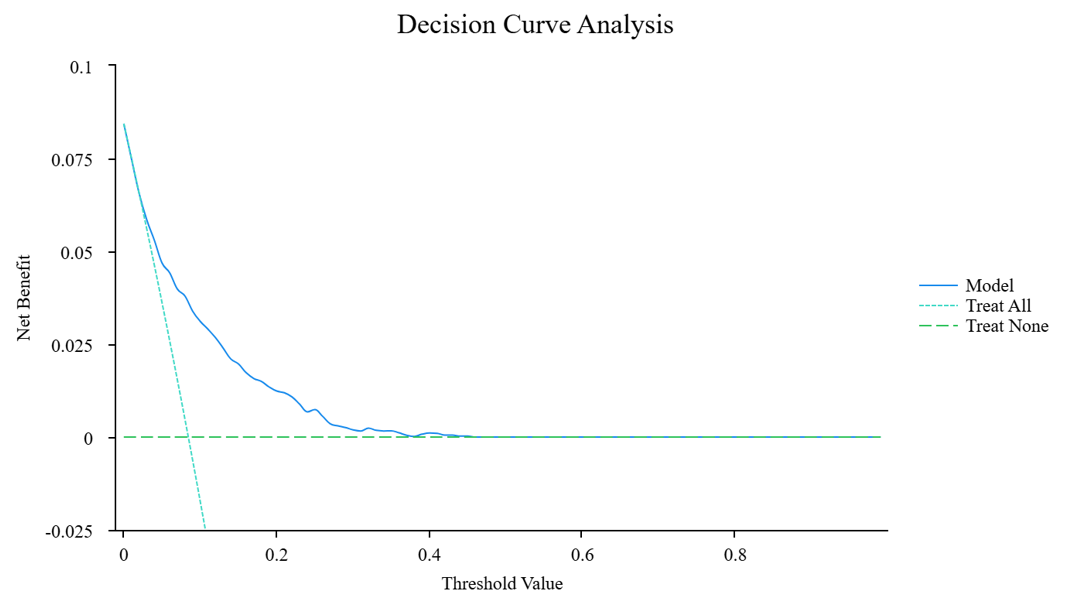
**

**Figure S5** DCA for AMD Risk Prediction by NHHR (NHANES)

**
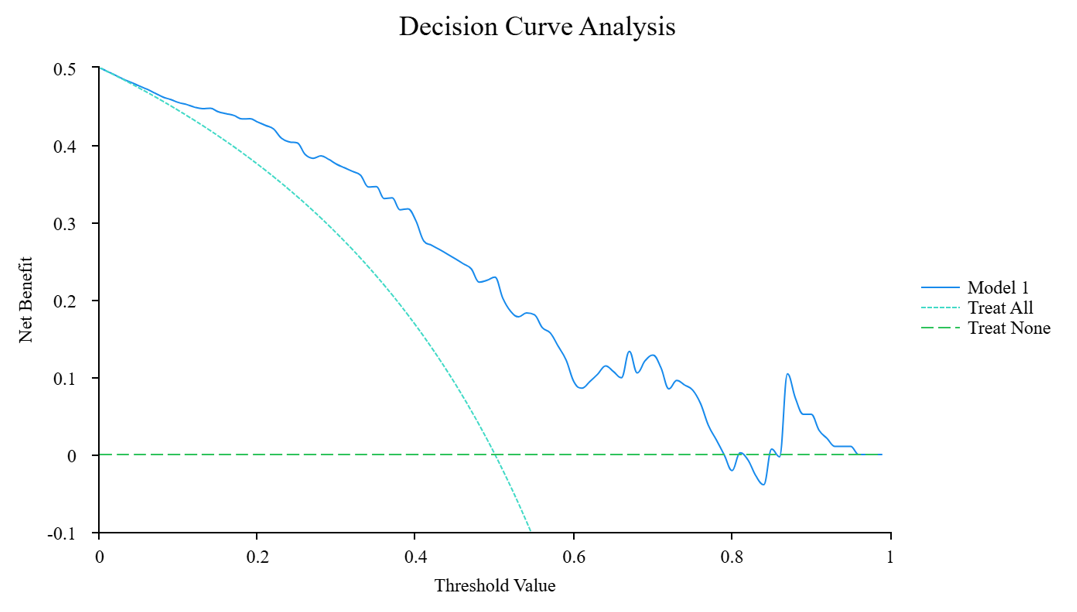
**

**Figure S6** DCA for AMD Risk Prediction by NHHR (PHFT)
