# Supplementary material for: Association between non-high-density lipoprotein cholesterol to high-density lipoprotein cholesterol ratio and age-related macular degeneration: insights from two observational studies
Source: Front Med (Lausanne). 2025 Dec 10;12:1724938. doi: 10.3389/fmed.2025.1724938 (PMC12728024; doi:10.3389/fmed.2025.1724938)
Supplement: Supplementary file 2 [file Data_Sheet_2.docx]

**Table S1** Associations between Non-HDL-C and its tertile and AMD among participants (NHANES)

| Variables | Non-adjusted OR(95%CI) | *P*-value | Adjust I  OR(95%CI) | *P*-value | Adjust II  OR(95%CI) | *P*-value |
| --- | --- | --- | --- | --- | --- | --- |
| Non-HDL-C | 0.99 (0.99, 1.01) | 0.024 | 0.99 (1.0,1.03) | 0.741 | 1.0(1.0, 1.0) | 0.699 |
| Non-HDL-C tertile | | | | | | |
| Q1 | 1.0(ref.) |  | 1.0(ref.) |  | 1.0(ref.) |  |
| Q2 | 0.88(0.65,1.19) | 0.409 | 1.10(0.79,1.55) | 0.554 | 1.12 (0.80,1.58) | 0.487 |
| Q3 | 0.75(0.52,1.08) | 0.116 | 0.96(0.66,1.41) | 0.836 | 0.96 (0.64,1.44) | 0.830 |
| *P* for trend | 0.87(0.73,1.03) | 0.108 | 0.98(0.82,1.78) | 0.856 | 0.98 (0.81,1.19) | 0.848 |

Model 0: no covariates were adjusted; Model 1: gender, age, and race were adjusted; Model 2: gender, age, race, marital status, BMI, smoking status, drinking status, diabetes, hypertension were adjusted. Abbreviation: AMD, age-related macular degeneration; BMI, body mass index; Non-HDL-C, non-high-density lipoprotein cholesterol; OR, odds ratio; 95% CI, confidence interval.

**Table S2** Associations between HDL-C and its tertile and AMD among participants (NHANES)

| Variables | Non-adjusted OR(95%CI) | *P*-value | Adjust I  OR(95%CI) | *P*-value | Adjust II  OR(95%CI) | *P*-value |
| --- | --- | --- | --- | --- | --- | --- |
| HDL-C | 1.01(1.003, 1.017) | 0.008 | 1.01 (1.0,1.02) | 0.040 | 1.01(1.0, 1.02) | 0.039 |
| HDL-C tertile | | | | | | |
| Q1 | 1.0(ref.) |  | 1.0(ref.) |  | 1.0(ref.) |  |
| Q2 | 1.10(0.880,1.373) | 0.391 | 1.02(0.80,1.31) | 0.842 | 1.03(0.79,1.33) | 0.827 |
| Q3 | 1.30(1.042,1.643) | 0.022 | 1.23(0.92,1.66) | 0.156 | 1.25(0.92,1.70) | 0.144 |
| *P* for trend | 1.15(1.018,1.289) | 0.025 | 1.11(0.96,1.30) | 0.16 | 1.12(0.96,1.30) | 0.144 |

Model 0: no covariates were adjusted; Model 1: gender, age, and race were adjusted; Model 2: gender, age, race, marital status, BMI, smoking status, drinking status, diabetes, hypertension were adjusted. Abbreviation: AMD, age-related macular degeneration; BMI, body mass index; HDL-C, high-density lipoprotein cholesterol; OR, odds ratio; 95% CI, confidence interval.

**Table S3** Associations between Non-HDL-C and its tertile and AMD among participants (PHFT)

| Variables | Non-adjusted OR(95%CI) | *P*-value | Adjust I  OR(95%CI) | *P*-value |  |  |
| --- | --- | --- | --- | --- | --- | --- |
| Non-HDL-C | 0.611 (0.398, 0.938) | 0.024 | 0.706 (0.444, 1.124) | 0.143 |  |  |
| Non-HDL-C tertile |  |  |  |  |  |  |
| Q1 | 1.0(ref.) |  | 1.0(ref.) |  |  |  |
| Q2 | 0.88(0.326,2.374) | 0.800 | 1.208(0.396,3.683) | 0.740 |  |  |
| Q3 | 0.358(0.130,0.989) | 0.048 | 0.499(0.164,1.518) | 0.220 |  |  |
| *P* for trend | 0.599(0.362,0.991) | 0.046 | 0.704(0.405,1.222) | 0.212 |  |  |

Model 0: no covariates were adjusted; Model 1: age and hypertension were adjusted. Abbreviation: AMD, age-related macular degeneration; Non-HDL-C, non-high-density lipoprotein cholesterol; OR, odds ratio; 95% CI, confidence interval.

**Table S4** Associations between HDL-C and its tertile and AMD among participants (PHFT)

| Variables | Non-adjusted OR(95%CI) | *P*-value | Adjust I  OR(95%CI) | *P*-value |  |  |
| --- | --- | --- | --- | --- | --- | --- |
| HDL-C | 4.666 (1.326, 16.42) | 0.016 | 9.879 (2.057, 47.44) | 0.004 |  |  |
| HDL-C tertile |  |  |  |  |  |  |
| Q1 | 1.0(ref.) |  | 1.0(ref.) |  |  |  |
| Q2 | 1.0(0.368,2.719) | 1.0 | 1.407(0.452,4.377) | 0.556 |  |  |
| Q3 | 1.735(0.649,4.637) | 0.272 | 2.793(0.885,8.814) | 0.080 |  |  |
| *P* for trend | 2.543(0.544,11.89) | 0.235 | 5.172(0.850,31.48) | 0.075 |  |  |

Model 0: no covariates were adjusted; Model 1: age and hypertension were adjusted. Abbreviation: AMD, age-related macular degeneration; HDL-C, high-density lipoprotein cholesterol; OR, odds ratio; 95% CI, confidence interval.

**Table S5** Diagnostic efficacy of ROC analysis of anthropometric indices(NHHR and its component lipids) for AMD(NHANES)

| Test | Best threshold | Accuracy | Sensitivity | Specificity | Postive predictive value | Negative predictive value | AUC(95%CI) |
| --- | --- | --- | --- | --- | --- | --- | --- |
| NHHR | 3.585 | 0.3368 | 0.7788 | 0.2961 | 0.0925 | 0.9356 | 0.539 (0.51,0.57) |
| Non-HDL-C | 144.5 | 0.5066 | 0.5664 | 0.5011 | 0.0947 | 0.9261 | 0.538(0.51,0.57) |
| HDL-C | 47.5 | 0.4309 | 0.6283 | 0.4127 | 0.0898 | 0.9234 | 0.521(0.49,0.55) |

**Table S6** Diagnostic efficacy of ROC analysis of anthropometric indices(NHHR and its component lipids) for AMD(PHFT)

| Test | Best threshold | Accuracy | Sensitivity | Specificity | Postive predictive value | Negative predictive value | AUC(95%CI) |
| --- | --- | --- | --- | --- | --- | --- | --- |
| NHHR | 3.0350 | 0.6562 | 0.5280 | 0.7917 | 0.7143 | 0.6230 | 0.674(0.56,0.78) |
| Non-HDL-C | 4.2250 | 0.6250 | 0.7708 | 0.4792 | 0.5968 | 0.6765 | 0.634(0.52,0.75) |
| HDL-C | 1.3100 | 0.6354 | 0.3958 | 0.8750 | 0.7600 | 0.5915 | 0.611(0.50,0.73) |

In the PHFT data, we constructed separate logistic-regression models for NHHR, Non-HDL-C, and HDL-C in relation to AMD to permit a direct comparison of the three indices. In unadjusted analyses, both NHHR (OR = 0.596, 95% CI: 0.413–0.861, *P* = 0.006) and non-HDL-C (OR = 0.611, 95% CI: 0.398–0.938, *P* = 0.024) showed statistically significant inverse associations with AMD, whereas HDL-C was significantly positively associated with AMD (OR = 4.666, 95% CI: 1.326–16.42, *P* = 0.016). These associations persisted after full adjustment (NHHR :OR = 0.592, 95% CI: 0.400–0.874, *P* = 0.008); HDL-C :OR = 9.879, 95% CI: 2.057–47.44, *P* = 0.004).

In the analysis based on NHHR tertiles, both models showed a significant negative correlation in the highest NHHR tertile (Model 0: OR = 0.238, 95% CI: 0.084–0.677, *P* = 0.007; Model 1: OR = 0.232, 95% CI: 0.072-0.750, *P* = 0.015). The results of trend analysis indicated that there was a significant dose-response relationship in both models (*P* values for trend were 0.008 and 0.015, respectively). In the unadjusted model, the highest tertile of Non-HDL-C and the trend test were statistically significant (OR = 0.238, 95% CI: 0.084–0.677, *P* = 0.007; *P* for trend = 0.046).However, after adjustment, neither the tertile-based Non-HDL-C model nor the full HDL-C model showed any significant associations (all *P* > 0.05) (Table 4–6).
